# Supplementary material for: Patterns of Intron Gain and Loss in Fungi
Source: PLoS Biol. 2004 Nov 30;2(12):e422. doi: 10.1371/journal.pbio.0020422 (PMC532390; doi:10.1371/journal.pbio.0020422)
Supplement: Table S1 — Also available at http://genes.mit.edu/NielsenEtAl/. (4.3 MB ZIP). [file pbio.0020422.st001.zip › NielsenEtAl/html/955.html]

AN3817.1.NCU00712.1.MG08975.1.FG09197.1


```
 CLUSTAL W (1.82) Multiple Sequence Alignments - Introns Inserted


Sequence 1: NCU00712.1	1173 aa
Sequence 2: MG08975.1	834 aa
Sequence 3: FG09197.1	1210 aa
Sequence 4: AN3817.1	1125 aa
Alignment Length: 1293 aa
Number Identitical Residues: 432 aa
Alignment Score (without introns) 22948


MG08975.1 	------------------------------------------------------------
NCU00712.1	MIASSLLPSRFRGEQPATQAATPSWINKKVTPPLQKLSKITSSNPIHTIVLVALLASSSY
FG09197.1 	-MASILLPKRFRGETAPAEKTTPSWASKRLTPIAQFISRLACSHPIHTVVLVAVLASTSY
AN3817.1  	-MASVLIRRKFGTE--GGSDAEPSWLKRQVTGCLQSISRRACIHPIHTIVVIALLASTTY
          	  ::         .    . : .:  .   :   .  :  :.  .  :    :  :::: 

MG08975.1 	------------------------------------------------------------
NCU00712.1	IGLLQNSLFN---VTR-SVRKAEWESLQAGSRMLRAGANTEWNWQNHDPEA--------P
FG09197.1 	VGLLQESFFS---TDLPTVGKADWSSLVEGSRVLRAGPETAWNWKAIEQDS--------I
AN3817.1  	VGLLEGSLFDSFRNSNNVAGHVDVDSLLLGNRSLRLGEGTSWKWQVEDSLNQDDQKVGNP
          	 .  . :  .:           . .:   ..     .  :  . .  .   ..... .. 

MG08975.1 	-----~------------------------------------------------------
NCU00712.1	VPA--~NANHLALLTLVFPDT--AESGPVVAQTNTVPIPSNLSITPLPSTAISFT-YSQD
FG09197.1 	QHAGA~DADHLALLTLVFPDTHSAESSSTAPRSSHVPVPQNLSITPLPSTKNSFTAYSQD
AN3817.1  	ELKRE0VDQHLALTTLIFPDS-ISKSASTAPAADAIPVPANASAQLLPHTPNLFSPFSHD
          	        .  :  :   ..:  :.:..  . :.  . . . :    . :    :. : .

MG08975.1 	--------------~---------------------------------------------
NCU00712.1	SALAFSLPYSQAPE~FLANAQEIPNAVSSQETIETERGHEKKMWIMKAARVQTRSSIVKW
FG09197.1 	SILAYSLPYAEGPD2-----------------------------------------VVQW
AN3817.1  	SSLVFTLPFDQVPQ~FLRAVQELPDPTLEDDEGEQKR---WIMRATRGPVSGPNGTISSW
          	:    : .  . ..      .. ...  ...  . .           ..   . .:  . 

MG08975.1 	-------------~----------------------------------------------
NCU00712.1	VQNAWVEFTDLLR~NAETLDIIIMALGYISMHLTFVSLFLSMRRMGSNFWLATSVIFSSI
FG09197.1 	ANNAWTEFLDLLK~NAETLDIVIMFLGYIAMHLTFVSLFLSMRKLGSKFWLGICTLFSSV
AN3817.1  	LSDAWSSFVDLIK0HAETIDIIIMTLGYLAMYLSFASLFFSMKQLGSKFWLATTVLFSGM
          	 ..:  .  .     :.: .      .  :   :  :   :    .:.   .     :. 

MG08975.1 	------------------------------------------------------------
NCU00712.1	FAFLFGLLVTTKLGVPMNMVLLSEGLPFLVVTIGFEKNIVLTRAVLSHAIDHRRPT--EK
FG09197.1 	FAFLFGLIVTTKLGVPISVILLSEGLPFLVVTIGFEKNIVLTRAVMSHAIEHRRQIQNSK
AN3817.1  	FAFLFGLLVTTKFGVPINILLLSEGLPFLVTTIGFEKPIILTRAVLSASIDKKRQG----
          	 :   .   ::. . . .    :.. .    : . ..    : :  : : .         

MG08975.1 	-------------------MAIKEKAYGILKDYAIEILILVCGAASGVQGGLQQFCFLAA
NCU00712.1	SGKPSKQADSAHSIQSAIQLAIKEKGFDIVKDYAIEAGILVLGAASGVQGGLQQFCFLAA
FG09197.1 	SGKGSPERSMQNVIQYAVQSAIKEKGFEIMRDYAIEIVILALGAASGVQGGLQHFCFLAA
AN3817.1  	-----SATSTPSSIQDSIQTAIREQGFEIIRDYCIEISILIAGAASGVQGGLRQFCFLAA
          	        .     . : . **:*:.: *::**.**  **  **********::******

MG08975.1 	WILFFDCILLFTFYTAILYIKLEINRIKRHVEMRKALEDDGLSRRVAENVASSN-DWPAG
NCU00712.1	WILFFDCILLFSFYTAILCIKLEINRIKRHVQMRKALEEDGVSRRVAEKVAQSN-DWPRA
FG09197.1 	WTLFFDFILLFTFYTAILSIKLEINRIKRHVDMRMALEDDGVSRRVAENVAKSDGDWTRV
AN3817.1  	WILFFDCLLLFTFYTTILCIKLEITRIRRHVTLRKALEEDGITQSVAEKVASSN-DWFGA
          	* **** :***:***:** *****.**:*** :* ***:**::: ***:**.*: **   

MG08975.1 	--KGSN---TSLFGKQIKSTSVPKFKGMMVSGFIIINLLNLCSIPFRSTHSLSSLSTWAG
NCU00712.1	--DGKDQPGTTIFGRQLKSTHIPKFKVMMVTGFVLINVLNLCTIPFRSANSISSISSWAR
FG09197.1 	--KGDS----SLFGR--KSSSVPTFKVLMILGFIFVNIVNICSIPFRNPRSLSTIRTWAS
AN3817.1  	GSDNSDADDASVFGRKIKSNNVRRFKFLMVGGFVLVNVVNMTAIPFRN----SSLSPLCN
          	.:....  .:::**:. **. :  ** :*: **:::*::*: :****.    *:: . . 

MG08975.1 	GLGGVLNSPPVDPFKVAANGLDEILGEAKSTNTETIVTILTPIKYELEYPSVHYALP---
NCU00712.1	GLGGVVTPPPVDPFKVASNGLDIILEAARADGRETIVTVLTPIRYELEYPSIHYDLP---
FG09197.1 	SLGGVVAPLSVDPFKVASNGLDAILAAAKSNNRPTLVTVLTPIKYELEYPSIHYALGSAI
AN3817.1  	----VFSPTPIDPFKVAENGLDAIYVSAKSQKLETLVTVVPPIKYKLEYPSVHYAKLG--
          	    *. . .:****** **** *   *::    *:**::.**:*:*****:**   .  

MG08975.1 	--PPKSAVP--PEADLGYGVGGRMVGGILKSLEDPVLSKWIVVALAMSVALNGYLFNAAL
NCU00712.1	--QKSAEVEGGDYANLG-GYGGRMVGSILKSLEDPILSKWIVVALALSVALNGYLFNAAR
FG09197.1 	NGNNAEYTDAFHHHFQGYGVGGRMVGGILKSLEDPVLSKWIVIALALSVALNGYLFNVAR
AN3817.1  	---ESQSIEIEYTDQLLDAVGGHVLNGVLKSIEDPVISKWIIAVLTLSIVLNGYLFNAAR
          	                  . **:::..:***:***::****: .*::*:.*******.* 

MG08975.1 	MGIKDPNAATQPINPRELADAQKFNDTDQAMLPLGEYQPPTAPPTPRRQPSPGVEEESDS
NCU00712.1	WGIKDPNVPDHPINPKELDEAQKFNDTASATLPLGEYMKPTAPSSPVAPLTPSSTDDEND
FG09197.1 	WGIKDPNVPEHNIDRNELARAQQFNDTGSATLPLGEYVPPTPMRT--EPSTPAITDDEAE
AN3817.1  	WSIKEPQAAPAPKEPAKPKVYPKIDLNAGPK-----------------------------
          	 .**:*:..    :  :     ::: .  .                              

MG08975.1 	GRTQ----VDNSPLPPAVPRTAAEIDTILKEKRVYELSDEEIVAYSLKGKIPGYSLERSL
NCU00712.1	AQAKENRAVTLAAQRATTIRSQGELDKMLAEKRTHELNDEEIVHLSLKGKIPGYALEKTL
FG09197.1 	GLQM-----TKARSDKLPNRPNEELEKLLAEKRVKEMSDEELVSLSMRGKIPGYALEKTL
AN3817.1  	-------------------RSMEECEAMLKAKKAAYLSDEELIELSLSGKLPGYALEKSL
          	                   *.  * : :*  *:.  :.***::  *: **:***:**::*

MG08975.1 	KD---------CTRAVKIRRTIISRTKATSSITSLLDRSKLPYRNYNWEQVLGACCENVI
NCU00712.1	KD---------FTRAVKVRRSIISRTKATTELTNILDRSKLPYQNYNWAQVHGACCENVI
FG09197.1 	GD---------FTRAVKIRRSIIARNRATSDLTHSLERSKLPFEKYNWERVFGACCENVI
AN3817.1  	ENEELMSRVDAFTRAVKIRRAVVSRTPATSAVTSSLETSKLPYKDYNYALVHGACCENVI
          	 :..  :  .: *****:**::::*. **: :*  *: ****:..**:  * ********

MG08975.1 	GFMPLPVGVAGPLVIDGQSYFIPMATTEGVLVASASRGCKAINAGGGAVTVLTGDGMTRG
NCU00712.1	GYMPLPVGVAGPLVIDGQSFFVPMATTEGVLVASTSRGCKAINSGGGAVTVLTADGMTRG
FG09197.1 	GYMPLPVGVAGPLVIDGQSYFIPMATTEGVLVASASRGCKAINAGGGAVTVLTADGMTRG
AN3817.1  	GTLPLPLGVAGPLVIDGQSYFIPMATTEGVLVASASRGAKAINAGGGAVTVLTGDGMTRG
          	* :***:************:*:************:***.****:*********.******

MG08975.1 	PCVGFETLERAGAAKNWLDSDVGQAFMTKAFNSTSRFARLESMKTAIAGTNLYIRFRTTT
NCU00712.1	PCVQFETLERAGAAKLWLDSEKGQSIMKKAFNSTSRFARLETMKTAMAGTNLYIRFKTTT
FG09197.1 	PCVAFETLERAGAAKLWLDSEAGSDIMKKAFNSTSRFARLQSMKTALAGTNLYIRFKTTT
AN3817.1  	PCVGFPTLARAAAAKVWLDSEEGKSVMTAAFNSTSRFARLQHLKTALAGTYLYIRFKTTT
          	*** * ** **.*** ****: *. .*. ***********: :***:*** *****:***

MG08975.1 	GDAMGMNMISKGVEEALRAMAAPDEGGFTDMQILTLSGNYCSDKKPAAINWIEGRGKSVV
NCU00712.1	GDAMGMNMISKGVEHALSVMYN---EGFEDMNIVSLSGNYCTDKKAAAINWIDGRGKSVV
FG09197.1 	GDAMGMNMISKGVEHALSVMSN--EAGFDDMQIVSVSGNYCTDKKAAALNWIDGRGKGVV
AN3817.1  	GDAMGMNMISKGVEKALHVMAT--ECGFDDMATISVSGNFCTDKKAAALNWIDGRGKSVV
          	**************.** .*    . ** **  :::***:*:***.**:***:****.**

MG08975.1 	AEAIIPGDIVRSVLKIDVDSLVEMNISKNLIGSAMAGAMGGFNAHAANIVAAVFLATGQD
NCU00712.1	AEAIIPADVVKNVLKTDVDTLVELNVNKNLIGSAMAGSMGGFNAHAANIVAAIFLATGQD
FG09197.1 	AEAIIPGDVVRSVLKSDVDALVELNISKNLIGSAMAGSVGGFNAHAANIVAAIFLATGQD
AN3817.1  	AEAIIPGDVVRNVLKSDVDALVELNTSKNLIGSAMAGSLGGFNAHASNIVTAIFLATGQD
          	******.*:*:.*** ***:***:* .**********::*******:***:*:*******

MG08975.1 	PAQVVESANCITIMRN2LRGNLQISVSMPSIEVGTLGGGTILEPQSAMLDLLGVRGSHPT
NCU00712.1	PAQVVESANCITLMRN2LRGNLQISVSMPSIEVGTLGGGTILEPQSAMLDMLGVRGPHPT
FG09197.1 	PAQVVESANCITIMKN2LRGALQISVSMPSLEVGTLGGGTILEPQSAMLDLLGVRGSHPT
AN3817.1  	PAQNVESSSCITTMKN2TNGNLQIAVSMPSIEVGTIGGGTILEAQGAMLDLLGVRGSHPT
          	*** ***:.*** *:*  .* ***:*****:****:*******.*.****:*****.***

MG08975.1 	TPGDNARRLARIIAAAVLAGELSLCSALAAGHLVKAHMAHNRSTPATRSTTPAPPATG~S
NCU00712.1	NPGENARRLARIVAAAVLAGELSLCSALAAGHLVKAHMAHNRSAPPTRTSTPAPAAA-~-
FG09197.1 	NPGDNSRRLARIIGASVLAGELSLCSALAAGHLVRAHMQHNRSAAPSRSTTPAPMTPV2R
AN3817.1  	NPGDNARQLARIVAAAVLAGELSLCSALAAGHLVRAHMAHNRSAAPTRSATPVSAAVG~-
          	.**:*:*:****:.*:******************:*** ****:..:*::**.. :    

MG08975.1 	GSGFMTPVGLAMTSAVEKATGGTLRSNKSSASLERQAAVGR-------------------
NCU00712.1	-------AGLTMTS-----------SNPNAAAVERSRR----------------------
FG09197.1 	----SFDTKVRCQPNNKDIRNILLTQHPSKPTITYSKRVIKSTIHLNPLILALFDNSVQT
AN3817.1  	--------------------------ATRGLSMTSSR-----------------------
          	                               ::  .                        

MG08975.1 	---------------------------------~-----
NCU00712.1	---------------------------------~-----
FG09197.1 	RDVQLGDQVSTRGTLDAVGGPQGGGVAAGGVAR0RVVGS
AN3817.1  	---------------------------------~-----
          	
```
